# Development and Characterization of a Guar Gum Bionanocomposite Loaded with Biogenic Selenium Nanoparticles and Its Cytotoxic Evaluation

**DOI:** 10.3390/gels12050376

**Published:** 2026-04-30

**Authors:** José Armando Hernández-Díaz, Ana Alejandra Arias-García, Alberto Gutiérrez-Becerra, Mauricio Comas-García, Milton Oswaldo Vázquez-Lepe, Orlando Hernández-Cristóbal, Soledad García-Morales, Moisés Martínez-Velázquez, Zaira Yunuen García-Carvajal

**Affiliations:** 1 Departamento de Biotecnología Médica y Farmacéutica, Centro de Investigación y Asistencia en Tecnología y Diseño del Estado de Jalisco A.C., Guadalajara 44270, Jalisco, Mexico; johernandez_al@ciatej.edu.mx (J.A.H.-D.); anarias_al@ciatej.edu.mx (A.A.A.-G.); 2 Departamento de Ciencias Básicas y Aplicadas, Centro Universitario de Tonalá (Cutonalá), Universidad de Guadalajara, Tonalá 45425, Jalisco, Mexico; alberto.gbecerra@academicos.udg.mx; 3 Centro de Investigación en Ciencias de la Salud y Biomedicina, Facultad de Ciencias, Universidad Autónoma de San Luis Potosí, San Luis Potosí 78210, Mexico; mauricio.comas@uaslp.mx; 4 Departamento de Ingeniería de Proyectos, Centro Universitario de Ciencias Exactas e Ingenierías (CUCEI), Universidad de Guadalajara, Guadalajara 44430, Jalisco, Mexico; milton.vazquez@academicos.udg.mx; 5 Escuela Nacional de Estudios Superiores, Universidad Nacional Autónoma de México, Morelia 58190, Michoacán, Mexico; microscopia@enesmorelia.unam.mx; 6 Departamento de Biotecnología Vegetal, Secretaría de Ciencia, Humanidades, Tecnología e Innovación-Centro de Investigación y Asistencia en Tecnología y Diseño del Estado de Jalisco A.C., Zapopan 45019, Jalisco, Mexico

**Keywords:** selenium nanoparticles, nanocomposites, guar gum, anticancer agents, cytotoxicity

## Abstract

Conventional chemotherapies for cervical cancer, such as cisplatin (CDDP)-based treatments, are limited by high systemic toxicity and the development of cellular resistance. To address these drawbacks, this study reports the green synthesis of selenium nanoparticles (SeNPs) using *Amphipterygium glaucum* leaf extract (AGLE) and the development of a guar gum-based nanocomposite (SeNPs@GG) loaded with these NPs. The synthesized SeNPs showed a stable UV–Vis absorption band at 275 nm, a spherical morphology, and sizes ranging from 11 to 21 nm, as confirmed by TEM. FTIR and XPS analyses demonstrated interactions between Se and functional groups from the plant extract, indicating its dual role as a reducing and stabilizing agent. The guar gum nanocomposites (NCs) exhibited a porous structure with a homogeneous distribution of SeNPs, as evidenced by SEM and EDS. At the same time, XRD confirmed the crystalline nature of the SeNPs. In vitro cytotoxicity assays using HeLa cervical cancer cells revealed significant antiproliferative effects with a biphasic response related to Se’s dual biological role. The IC_50_ values were 98.3 µg/mL for SeNPs, 93.7 µg/mL for SeNPs@GG1, and 93.5 µg/mL for SeNPs@GG2. Additional analyses confirmed apoptosis, DNA fragmentation, ROS production, mitochondrial dysfunction, and G2/M cell cycle arrest, supporting the potential of these systems as alternative chemotherapeutic strategies.

## 1. Introduction

Selenium (Se) is an essential element for life and is constitutively present in mammals; it is involved in key biological functions, such as immune regulation and protection against reactive oxygen species (ROS)-induced cellular damage, through its antioxidant activity. It has been reported that Se, at low concentrations, participates in the activity of enzymes such as glutathione peroxidase (GPx). In contrast, at higher concentrations, it may induce cytotoxicity and apoptosis in tumor cells [1]. This has generated interest in its application as an anticancer agent. However, the use of Se in ionic forms, such as sodium selenite (Na_2_SeO_3_), is limited by significant non-selective systemic toxicity, which restricts its therapeutic use [2].

In this context, the development of SeNPs has emerged as a promising alternative due to their improved bioavailability and reduced toxicity. Likewise, they may exhibit greater specificity for tumor cells than ionic Se does. In addition, SeNPs can activate multiple mechanisms of cell death, thereby increasing their versatility as antitumor agents [3]. The synthesis of these types of nanoparticles (NPs) using plant extracts represents a sustainable and biocompatible strategy, further reducing toxicity for biological applications and avoiding the use of toxic reducing or stabilizing agents commonly employed in conventional chemical methods [4].

In particular, the leaf extract of *A. glaucum*, an endemic species of Western Mexico in the genus *Amphipterygium* and family Anacardiaceae, exhibits various biological activities, including antioxidant, antimicrobial, and anticancer properties [5]. The genus *A. glaucum* belongs to a group of plants with a long-standing history in traditional medicine since pre-Hispanic times, where ethnopharmacological knowledge has guided the identification of bioactive compounds with therapeutic potential, including anticancer activity [6]. Despite this relevance, *A. glaucum*, like approximately 90% of Mexican medicinal plant species, remains insufficiently studied from phytochemical and pharmacological perspectives [6], as well as in the context of plant-mediated nanoparticle synthesis and its applications. Therefore, incorporating *A. glaucum*, a poorly characterized species, into this framework contributes not only to expanding the repertoire of plant-based nanomaterial synthesis but also to uncovering new biofunctional nanocomposites with potential applications. *A. glaucum* also shows potential as a natural reducing and stabilizing agent for NPs synthesis, promoting the formation of NPs with enhanced bioactive properties and more acceptable safety profiles for biomedical applications [7].

The use of biological agents for nanoparticle (NP) synthesis is generally considered less efficient than chemical approaches. Therefore, to improve NP stability, dispersion, and controlled release, their incorporation into synthetic or natural polymer matrices has been widely explored [8]. Synthetic polymers such as polyvinyl alcohol (PVA) and polyvinylpyrrolidone (PVP) stabilize the SeNPs [9,10,11].

On the other hand, polysaccharides such as guar gum (GG) not only act as colloidal stabilizers but can also form hydrogels or NCs that protect NPs from aggregation and enable controlled release of the active compound [12]. GG is a natural, biodegradable, and non-toxic polysaccharide [13] and has been used as an efficient surface coating agent in the synthesis of metallic NPs [14], as a reducing agent and stabilizing matrix for silver nanoparticles synthesis [15], or as a matrix in which selenite salt is embedded for cardiac problems [14,16,17].

One of the clinical contexts in which SeNPs have shown the most promising results is cancer, specifically cervical cancer [18], the fourth most common cancer among women worldwide, and a significant cause of mortality in developing countries [19]. SeNPs have demonstrated the ability to induce cell death in cervical cancer cells. In vitro studies using the HeLa cell line have shown that, through mechanisms such as ROS generation, mitochondrial damage, and DNA fragmentation, cancer cells can be eliminated with minimal impact on non-tumor cells [18].

Although guar gum has been widely used as a stabilizing agent for SeNPs, and *Amphipterygium* extracts have been employed for green nanoparticle synthesis, the combination of biogenic SeNPs synthesized using *A. glaucum* leaf extract and subsequently embedded in a guar matrix has not been described before this study. To the best of our knowledge, this is the first report to describe the incorporation of biogenic SeNPs into a guar gum-based matrix prepared by freeze/thaw and subsequent lyophilization, along with a systematic in vitro safety evaluation in HeLa cells. This work bridges green nanoparticle synthesis and polysaccharide-based biomaterials, providing a novel platform for developing functional SeNP-loaded systems.

## 2. Results and Discussion

### 2.1. Synthesis of SeNPs and Nanocomposites (NCs)

**Figure 1:**
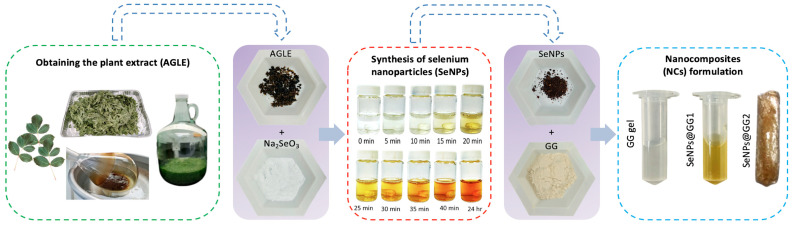
Schematic overview of the synthesis of SeNPs and fabrication of guar gum-based nanocomposites (NCs), including *A. glaucum* extract preparation, color changes during synthesis, and pre- and post-lyophilization NC forms.

The green synthesis of SeNPs was achieved by chemical reduction in Na_2_SeO_3_ using the methanolic extracts of *A. glaucum* as both reducing and stabilizing agents. After that, the SeNPs were embedded in a GG polymer matrix to enhance their physical and chemical properties. During the SeNPs synthesis process, a color change was observed in the solution, from colorless to light yellow, then to reddish-brown (Figure 1). This variation in the colouration of colloidal Se may suggest an increase in the size of the formed SeNPs. Likewise, color changes may be attributed to the excitation of surface plasmon vibrations or surface plasmon resonance (SPR) of the SeNPs, where the interaction of light with the free electrons on the surface of the NPs generates different hues depending on the size, shape, and composition of the material, providing evidence of SeNPs formation [20].

Significant differences in size have been reported depending on coloration, with more homogeneous NPs. In addition, it has been demonstrated that these characteristics are influenced by the synthesis method used. For example, when chemical compounds such as sodium thiosulfate pentahydrate (Na_2_S_2_O_3_·5H_2_O) are employed as reducing agents, the resulting SeNPs exhibit average sizes of 20 nm and highly homogeneous spherical morphologies [21]. Similarly, the use of ascorbic acid as a reducing agent has been shown to yield spherical NPs with homogeneous sizes of 40–60 nm [22].

Conversely, the use of biological sources for SeNPs synthesis has been shown to yield greater variability in results and larger NPs. This is attributed to the presence of biomolecules coating the NPs, which modify their optical properties and give them a brownish-red appearance [22]. This information has been corroborated by Garza et al. [7], who, using extracts from the same species, reported the formation of semi-spherical SeNPs with colorations similar to those obtained in this study. Other studies have also reported color changes during NPs formation, with brownish-red hues predominating in syntheses using plant extracts such as *Gymnanthemum amygdalinum*, *Malpighia emarginata*, and *Allium* [23].

The stability of colloidal systems at the nanoscale is a key factor for biomedical applications. The synthesized SeNPs were incorporated into a GG polymeric suspension and subsequently lyophilized to obtain a porous polymer matrix, readily dispersible in water, similar to those previously described for PVA porous matrices loaded with SeNPs [9].

GG is a biocompatible polymer that provides suitable protection sites to minimize NPs aggregation and stabilize the colloidal suspension [24]. Moreover, processes such as lyophilization have proven effective strategies for preserving the properties and efficacy of NPs over long periods [25]. GG has been widely used in nanoformulations to enhance drug delivery and improve the stability of nanostructured systems [26,27]. Hasan et al. [28] also formulated a polymeric matrix using GG that successfully maintained NPs’ stability. Other natural polymers, such as pectin, have similarly been shown to stabilize NPs within NCs [29].

The synthesis of SeNPs yielded a high gravimetric recovery (99.56% ± 0.29), indicating negligible mass loss during processing and lyophilization. However, when the yield was calculated on a stoichiometric basis considering the theoretical selenium content derived from sodium selenite, a value of 218.07% ± 0.64 was obtained. This apparent yield exceeding 100% suggests that the recovered material is not exclusively composed of elemental selenium but may also contain plant-derived organic compounds and residual selenium species [30,31]. Indeed, it has been reported that selenium can exist in multiple oxidation states during NPs’ synthesis, and that incomplete reduction or residual precursor species may persist even after purification, contributing to the overall composition of the final material [31]. Notably, consistent yield values across independent batches (*n* = 4) further support the reproducibility and robustness of the SeNPs nanocomposites’ preparation process.

In our synthesis, we used ~10 mM sodium selenite, comparable to that reported by Soliman et al. [32], who used a synthesis based on *Cassia javanica*-based synthesis (180 mL, 10 mM sodium selenite). Dos Santos Souza et al. [23], who used a combination of plant extracts (onion, acerola, boldo) and employed 10–20 mM selenium salt [23]. Despite the significantly lower sodium selenite dose used in this study, the precursor concentration remains consistent across SeNPs synthesis. Notably, differences in reaction time, extract composition, and processing conditions suggest that variations in yield and nanoparticle properties are primarily governed by the nature of the reducing agents and synthesis parameters rather than precursor concentration alone.

### 2.2. Physicochemical Characterization of SeNPs and NCs

#### 2.2.1. UV–Visible Spectroscopy

Key parameters, including reaction time, pH, temperature, precursor concentration, and plant extract concentration, strongly influenced the formation of SeNPs. SeNPs were optimally synthesized at pH 10.7, 45 °C, 10 mM Na_2_SeO_3_, 5% AGLE, and 24 h of reaction time. These conditions favored the complete reduction of Se^4+^ to Se^0^, and the careful control of these parameters is essential for reproducible and scalable SeNPs synthesis [33].

**Figure 2:**
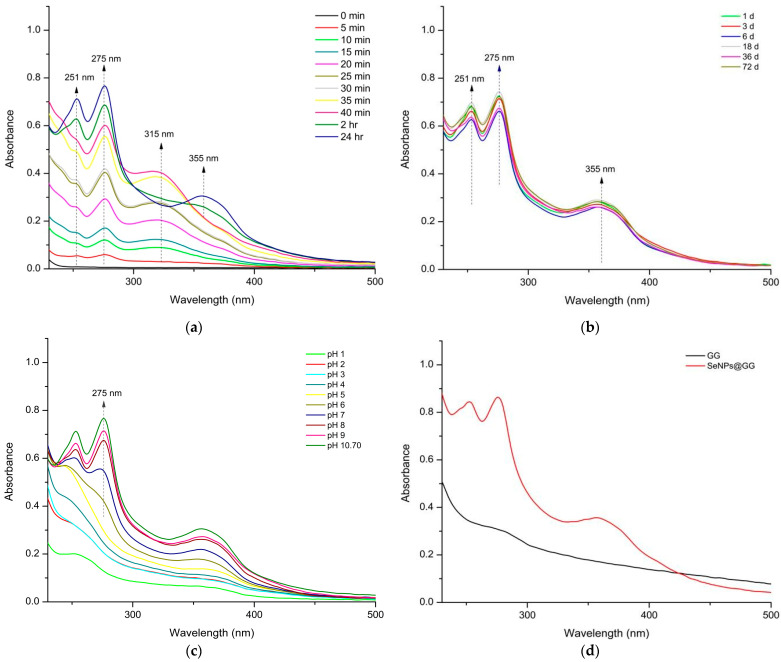
Synthesis and characterization of SeNPs. (**a**) Formation of SeNPs and UV–Vis absorbance spectra from 0 to 24 h; (**b**) UV–Vis spectra showing SeNPs stability over time; (**c**) stability analysis of SeNPs at different pH values; (**d**) absorbance spectra of SeNPs within (NCs) and pure (GG).

UV–Vis spectroscopy was used to monitor the formation and evolution of selenium nanoparticles (SeNPs) in the 200–500 nm range. As shown in Figure 2a, the spectra exhibited three reproducible absorption features centered at ~251, ~275, and ~355 nm. The band near 270–280 nm is consistent with previous reports on biologically synthesized SeNPs, where a broad absorption maximum in this region is commonly used as an early indicator of nanoparticle formation [4,9,34].

This band appeared within the first minutes of reaction and remained detectable over time, suggesting a rapid reduction in the selenium precursor by the plant extract. The features observed at ~251 and ~355 nm likely arise from a combination of nanoparticle-associated transitions and the presence of residual phytochemicals [4,34,35,36]. Organic molecules from plant extracts exhibit strong absorbance in the 230–330 nm region and can remain associated with SeNPs, influencing the shape and position of the UV–Vis bands [4,34].

Therefore, while the 270–280 nm feature supports nanoparticle formation, the overall spectrum must be interpreted as the result of both SeNPs and extract-derived chromophores. Stability studies conducted over 72 days at 5 °C showed that the absorption band near 270 nm retained its intensity with minimal variation (Figure 2b), indicating good colloidal stability under alkaline synthesis conditions. The persistent presence of the SeNPs band at basic pH has been reported in other biogenic systems and is commonly attributed to the deprotonation of capping biomolecules, thereby enhancing electrostatic stabilization [37]. In contrast, gradual acidification of the dispersion produced a marked decrease and eventual disappearance of the ~270 nm band (Figure 2c). This behavior aligns with observations from other SeNPs’ syntheses, in which acidic environments lead to protonation of surface functional groups, loss of electrostatic repulsion, and nanoparticle aggregation, accompanied by a collapse of the UV–Vis signal [38].

In the nanocomposites (NCs), the characteristic absorption near 270 nm remained detectable after incorporating SeNPs into the guar gum (GG) matrix and subsequent lyophilization (Figure 2d). Pure GG exhibited a broad, featureless absorption in the 280–300 nm region, in agreement with previous reports on polysaccharide-based materials [39]. The presence of the ~270 nm band in the NCs, absent in pure GG, supports the retention of SeNPs within the polymer matrix. Nevertheless, because GG and plant-derived compounds also absorb in the UV region, minor spectral overlap cannot be fully excluded.

#### 2.2.2. FTIR Spectrum Analysis

FTIR spectroscopy was used to identify the functional groups present in AGLE to clarify its role in reducing Na_2_SeO_3_ ions. Spectra of the extract, SeNPs, and SeNPs@GG were analyzed in the 4000–500 cm^−1^ region. The observed bands allowed inference of interactions between extract compounds and Se, supporting its reducing capacity and stabilizing function through binding to the surface of SeNPs. A broad and intense band at 3310 cm^−1^ was identified, attributed to the stretching of –OH groups in alcohols and phenols, consistent with other studies using plant extracts in NPs’ synthesis [40].

**Figure 3:**
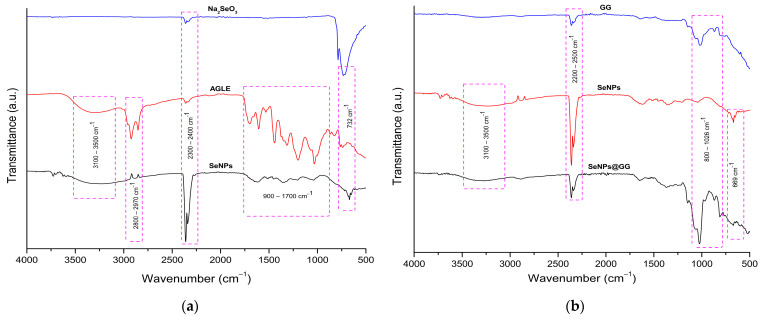
FTIR-ATR spectra of (**a**) Na_2_SeO_3_, *A. glaucum* leaf extract (AGLE), and SeNPs; (**b**) guar gum (GG), SeNPs, and the nanocomposite (SeNPs@GG).

The FTIR analysis revealed significant shifts in the functional bands following AGLE-mediated SeNPs’ synthesis, indicating key chemical interactions during NPs’ formation and stabilization (Figure 3a). A shift in the band associated with –OH stretching was observed, moving from 3310 cm^−1^ in the extract to 3277 cm^−1^ in the SeNPs, suggesting the involvement of hydrogen bonding interactions between Se and phenolic or alcoholic hydroxyl groups. Additionally, the bands at 2923 and 2852 cm^−1^, attributable to C–H vibrations of alkane-type structures, monoterpenes, and carboxylic acids, may confirm the participation of these compounds in reduction and stabilization processes [41].

A strong signal at 2350 cm^−1^ was also detected in the SeNPs, possibly associated with triple-bond vibrations (C≡C, C≡O, or C≡N), whose intensity increased notably compared with the extract. The carbonyl bands observed at 1700 and 1605 cm^−1^ in the extract shifted to 1624 and 1510 cm^−1^ in the SeNPs, respectively, suggesting interactions between C=O groups and amides with the NPs’ surface. Other representative bands corresponding to C–C stretching of aromatic compounds, C–O, and C–N, originally located at 1443, 1313, and 1199 cm^−1^, shifted to 1438, 1352, and 1207 cm^−1^, indicating the formation of Se–O bonds [7]. The band at 1048 cm^−1^ (C–OH), associated with alcohols, was particularly relevant, shifting to 1037 cm^−1^, indicating direct interaction with reduced Se. Moreover, the signal corresponding to pure Na_2_SeO_3_ at 732 cm^−1^ was observed at 669 cm^−1^ in the SeNPs, suggesting the formation of Se–O bonds through metal–oxygen stretching vibrations [42].

In the NCs (Figure 3b), the FTIR spectra retained the characteristic bands of the SeNPs, reflecting their post-synthesis incorporation into the polymeric matrices. However, interactions between the SeNPs and the polymer were evidenced by shifts or decreases in band intensity. For SeNPs@GG, the broad band between 3100 and 3500 cm^−1^ shifted to higher frequencies and broadened, indicating modifications in intra- and intermolecular hydrogen bonding. Likewise, an increased intensity of the 1026 cm^−1^ band was observed in SeNPs@GG, attributable to polysaccharide ring vibrations and side groups (C–OH) [43]. Finally, the presence of bands near 800 cm^−1^, characteristic of galactomannans associated with α and β anomeric configurations and glycosidic linkages attributed to α-D-galactopyranose and β-D-mannopyranose units, confirmed the interaction of the polymer with the NPs in the fingerprint region [27].

#### 2.2.3. X-Ray Photoelectron Spectroscopy Analysis

**Figure 4:**
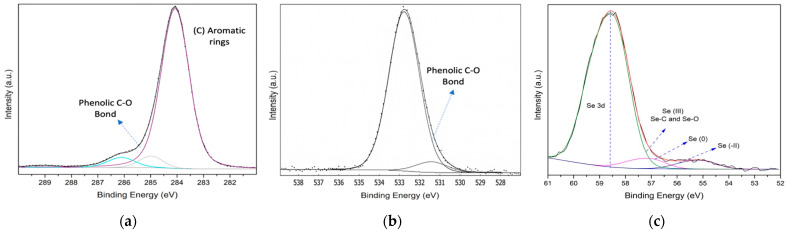
High-resolution XPS spectra showing (**a**) C1s and (**b**) O 1s signals of the AGLE and (**c**) Se 3d spectra of SeNPs.

X-ray photoelectron spectroscopy (XPS) was used to characterize the outermost surface to determine the chemical specificity of the elements present in the AGLE and the SeNPs. The spectra at high resolution showed a carbon C1s mean peak at 284.5 eV (Figure 4a), which is associated with CH and C=C bonds, mainly due to phenolic groups and aromatic bonds. Minor peaks at 285.2 eV are associated with the CH–CH bonds, and 286.3 eV of binding energy is attributed to C–O bonds in the hydroxyl group, in the plant extract. The high-resolution spectra of oxygen O1s (Figure 4b) show a main peak at a binding energy of 532.6 eV, which is attributed to C–OH in alcohol form or C–O–C in either form, as a typical binding energy for organic samples. The minor component at 531.4 eV can be related to the C=O carbonyl group, an oxygen–carbon bond attached to the aromatic structure.

It has been demonstrated that the selenium Se3d signal consists of a doublet by the spin-orbit, 3d3/2 and 3d5/2 [44]. Using an area ratio of 0.6867 for deconvolution, the high-resolution analysis of the Se3d spectrum in SeNPs revealed three components (Figure 4c). The elemental selenium (Se^0^) signal is expected to appear at 55.3 eV in this case, a minor component. Another component at 57.0 eV corresponds to Se(II) species, likely arising from redox processes involving extract compounds, as an intermediate ion (SeO_2_) and finally the signal at 58.3 eV the main signal is attributed to Se(IV) [45], suggesting the formation of Se–O and Se–C bonds through interactions with functional groups in the extract, a common phenomenon in biogenically synthesized or extract-capped SeNPs. These findings are consistent with previous reports, such as that by Ruiz-Fresneda et al. [46], which identified similar signals in the 55–58 eV region for biogenically synthesized SeNPs.

#### 2.2.4. Transmission Electron Microscopy Analysis

**Figure 5:**
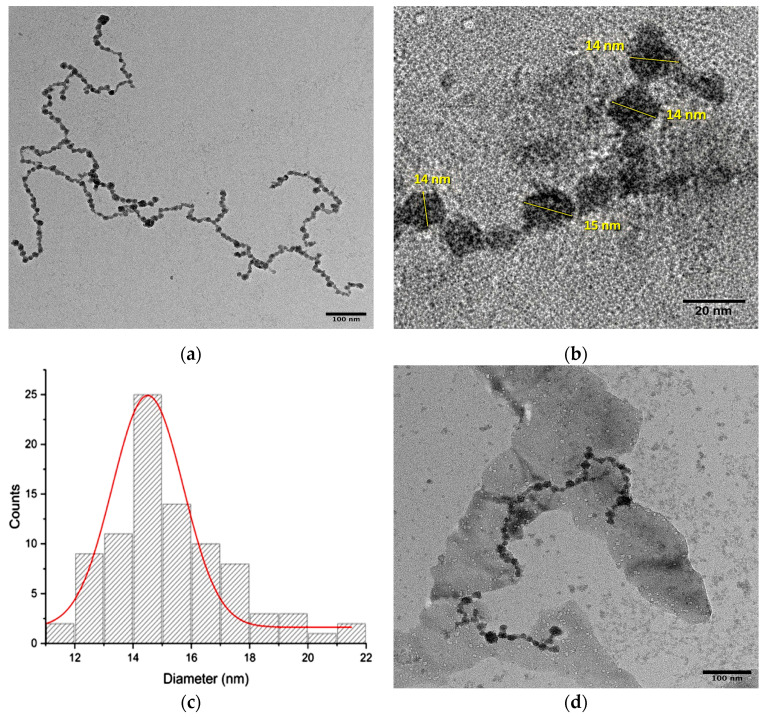
TEM micrographs of SeNPs and nanocomposites. (**a**,**b**) High-resolution images of SeNPs; (**c**) particle size distribution; (**d**) micrograph of SeNPs@GG.

Transmission electron microscopy (TEM) was used to confirm the morphology and size of the SeNPs synthesized from AGLE. The micrographs revealed a polydisperse distribution of predominantly semi-spherical SeNPs (Figure 5a,b), with diameters ranging from 11 to 21 nm (Figure 5c). The particles were observed embedded in an amorphous network of phytochemical compounds, presumably originating from the extract, suggesting potential interactions between organic functional groups and the SeNPs surfaces. These interactions could promote the observed partial aggregation, a phenomenon supported by molecular dynamics studies that link the properties of organic ligands to nanoparticle aggregation.

In the micrographs of the nanocomposite (SeNPs@GG), the successful incorporation of SeNPs into the polymeric matrix was confirmed (Figure 5d). The particles retained their semi-spherical morphology and an average size of ~14 nm, exhibiting branching-type clustering, possibly induced by the phytochemical coating surrounding the NPs. Polymer agglomerates physically encapsulating the SeNPs were also visible. These findings are consistent with previous studies reporting similar morphological characteristics when synthesizing SeNPs with other plant extracts [47].

#### 2.2.5. Scanning Electron Microscopy Analysis

**Figure 6:**
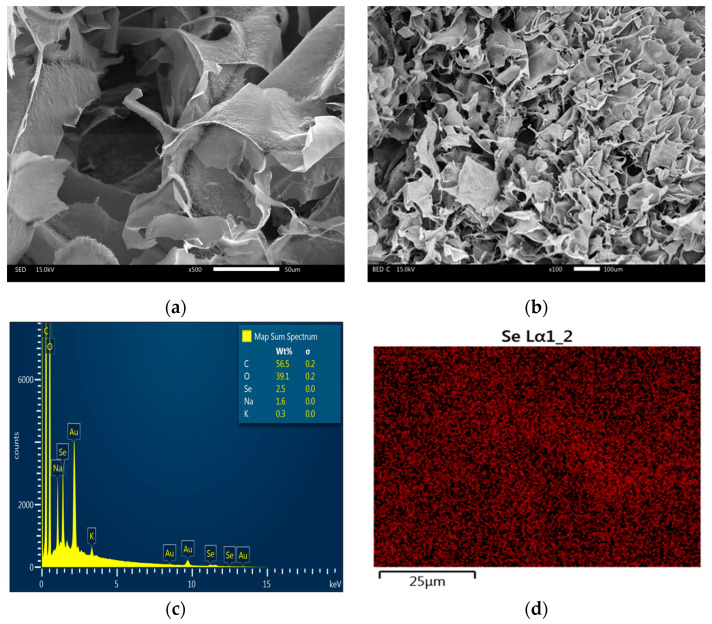
SEM analysis of SeNPs@GG. (**a**,**b**) Surface morphology of guar gum-based NCs; (**c**) EDS spectrum of the NCs; (**d**) elemental mapping of Se by EDS.

The morphological characterization of the NCs (SeNPs@GG) was performed using scanning electron microscopy (SEM) and EDS microanalysis for elemental mapping. The micrographs revealed that the NCs primarily exhibited laminar architecture with limited interconnections, comprising thin, heterogeneous walls (Figure 6a,b).

It was not possible to observe the SeNPs, likely due to their small size or their possible embedding within the polymeric matrix. EDS analysis reported 2.5% Se (Figure 6c). In addition, EDS elemental mapping revealed a homogeneous distribution of Se within the NCs (Figure 6d), confirming that the method for incorporating SeNPs into the NCs was adequate. These results are consistent with those reported by Modrzejewska-Sikorska et al. [48], who used SeNPs mapping to analyze the homogeneous distribution of Se.

#### 2.2.6. X-Ray Diffraction Analysis

**Figure 7:**
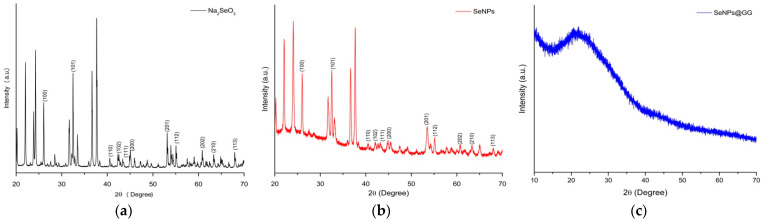
X-ray diffraction (XRD) patterns of (**a**) Na_2_SeO_3_, (**b**) SeNPs synthesized using *A. glaucum* leaf extract (AGLE), and (**c**) guar gum nanocomposite containing SeNPs (SeNPs@GG).

X-ray diffraction (XRD) analysis was used to characterize the crystalline structure of the SeNPs synthesized by AGLE (Figure 7). The results revealed that the SeNPs (Figure 7b) exhibited a diffraction pattern characteristic of crystalline materials, comparable to that observed for Na_2_SeO_3_ (Figure 7a). Eleven well-defined peaks were identified in the XRD spectrum at 2θ angles of 26.75°, 32.45°, 40.34°, 42.14°, 43.06°, 45.09°, 53.76°, 55.22°, 62.34°, 64.87°, and 69.14°, corresponding to the crystallographic planes (100), (101), (110), (102), (111), (200), (201), (112), (202), (210), and (113). These values are consistent with those reported in the JCPDS card No. 06–0362 for powdered Se, confirming the crystalline phase and structural purity of the synthesized SeNPs [49]. Similarly, biogenically synthesized NPs from *Alcaligenes faecalis* exhibited diffraction patterns characteristic of Se, consistent with those of the NPs synthesized in this study [50]. Likewise, peak broadening was observed in the SeNPs diffractogram relative to that of Na_2_SeO_3_. This behavior is attributable to nanoparticle formation, as crystallite size decreases from bulk to nanoscale dimensions [51].

These findings are consistent with those reported by Meenambigai et al. [52], who successfully synthesized SeNPs with a well-defined crystalline structure using leaf extracts of *Nilgirianthus ciliatus*, yielding diffraction patterns similar to those observed in this study, thereby confirming the nanocrystalline structure. Conversely, the structural analysis of SeNPs stabilized with GG (SeNPs@GG) showed a different pattern (Figure 7c), as GG exhibits a predominantly amorphous pattern with low crystallinity. However, an ordered region was detected at 2θ = 21.54°. This observation is in agreement with previous reports describing crystalline regions in GG-based polymeric matrices at (2θ) = 20.2° and 22.6° [27,53]. The incorporation of amorphous polymers, such as GG, into NC formulations can modify nanocrystalline structure formation [54]. Furthermore, several studies have demonstrated that the crystalline structure of NPs can significantly influence their physicochemical and biological properties [55]. Variations in crystalline order directly affect surface characteristics, such as surface energy, charge, solubility, and protein interactions, which, in turn, determine their behavior in biological systems and subsequent cellular responses [56].

### 2.3. In Vitro Evaluation of Cytotoxic Activity

The cytotoxic activity of SeNPs, Na_2_SeO_3_, extract (AGLE), GG, and the NCs, SeNPs@GG1 (liquid, non-lyophilized) and SeNPs@GG2 (lyophilized and re-dissolved), was evaluated by determining cell viability, programmed cell death, reactive oxygen species generation (ROS), cell morphology, morphological alterations in the cell membrane, integrity of the mitochondrial membrane potential, DNA fragmentation, cell cycle modifications, and migratory capacity in the HeLa cells model.

#### 2.3.1. Cellular Viability by MTT Assays

**Figure 8:**
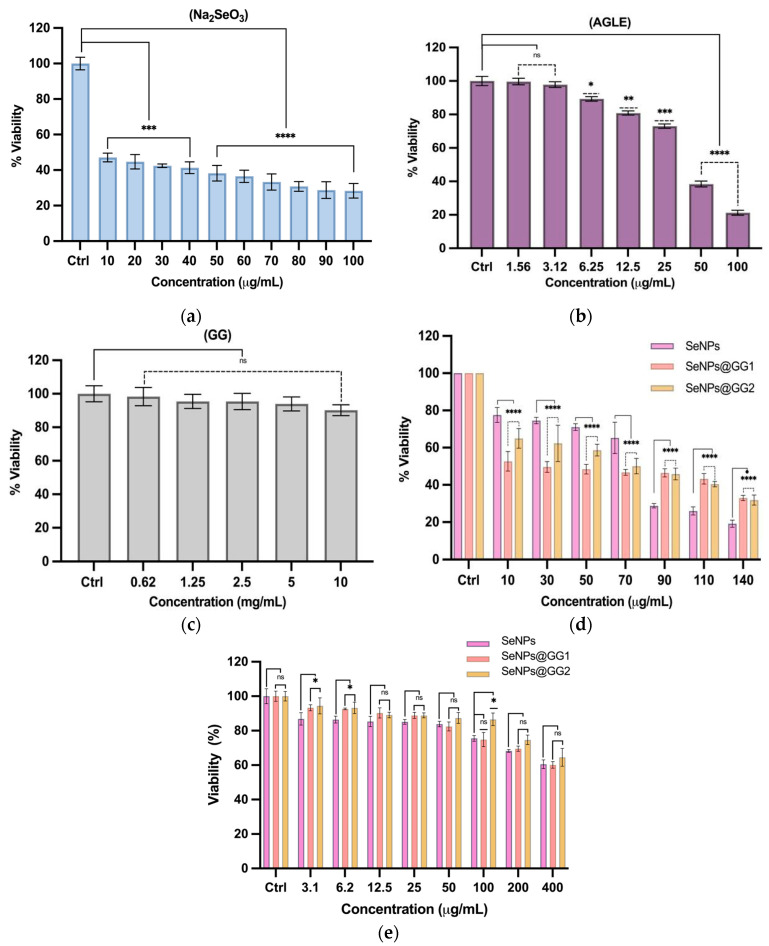
Cytotoxicity evaluation in HeLa cells by MTT assay. (**a**) Na_2_SeO_3_ treatment (24 h); (**b**) AGLE treatment; (**c**) cell viability after GG exposure (0.6–10 mg/mL); (**d**) treatments with SeNPs, (SeNPs@GG1 and SeNPs@GG2) at 10–140 µg/mL; (**e**) treatments with SeNPs, (SeNPs@GG1 and SeNPs@GG2) at 3.1–400 µg/mL, on primary endometrial cells. Data are expressed as mean ± standard deviation. Statistical significance was determined by one- or two-way ANOVA (* *p* < 0.05, ** *p* < 0.01, *** *p* < 0.001, **** *p* < 0.0001), ns: not significant.

Cell viability was assessed 24 h post-treatment using the MTT assay. The concentrations evaluated ranged from 1.5 to 140 µg/mL for most compounds, except for GG, which was tested at 0.6–10 mg/mL. Na_2_SeO_3_ (Figure 8a) exhibited a marked cytotoxic profile, reducing cell viability by approximately 50% from the lowest concentration tested, with an IC_50_ of 40.2 µg/mL. This result suggests that ionic selenium species are more toxic than Se in nanoparticulate form, as previously reported [57].

The AGLE exhibited a significant reduction in cell viability, with an IC_50_ value of 26.6 µg/mL (Figure 8b). This effect may be related to the presence of triterpenic compounds in *A. glaucum*, including masticadienonic acid, 3α-hydroxymasticadienonic acid, and 3β-hydroxymasticadienonic acid. Gómez-Cansino et al. [58] reported that these metabolites are responsible for the extract’s antiviral activity, with an IC_50_ of 97.8 µg/mL. Additionally, anticancer activity has been documented in extracts of *A. adstringens*, a species of the same genus used in this study, where anacardic acids such as 6-nonadecenyl salicylic acid (C19:0) have been identified. Oral administration of this compound at 10 mg/kg in murine models resulted in cytotoxicity in peripheral blood cells [59].

In contrast, GG did not exhibit significant cytotoxicity, maintaining cell viability above 90% even at concentrations up to 10 mg/mL (Figure 8c). This behavior is expected, as GG is a natural, biocompatible, and inert polysaccharide with no reactive groups that could damage proteins, lipids, or DNA. Its high molecular weight and hydrophilic nature prevent cellular uptake via endocytosis, thereby avoiding intracellular toxic interactions. Moreover, in solution or gel form, GG does not alter medium pH nor generate harmful by-products [60].

On the other hand, the SeNPs reduced cell viability to below 80% starting at the lowest tested concentration. However, they displayed non-linear behavior, with a plateau between 10 and 70 µg/mL, during which cell viability remained stable. At higher concentrations, a sharp increase in cytotoxicity was observed, reaching an IC_50_ of 98.3 µg/mL (Figure 8d), consistent with previous reports [9]. This biphasic behavior has been described in normal fibroblasts (L929) and human glioblastoma cells (U87) and is attributed to Se’s dual role: an antioxidant at low concentrations and a cytotoxic agent at higher doses [61]. Several studies highlight this concentration-dependent antioxidant/pro-oxidant behavior, showing that Se may act as an antioxidant at low levels, mainly through enzymes such as glutathione peroxidase (GPx). At high concentrations, it induces reactive oxygen species (ROS), surpassing cellular antioxidant capacity, and triggering cell death [62]. The plateau behavior may also be related to the physicochemical properties of SeNPs, whose slower release rate compared to ionic Se could delay toxicity. Likewise, the formation of protein coronas around NPs or the saturation of Se transporters, such as SELENOP receptors, ApoER2 (LRP8), megalin (LRP2), and other LRPs, could temporarily modulate cellular uptake, leading to the observed viability plateau [63,64]. In vivo studies have shown that low doses of sodium selenite (0.2 mg/kg diet) improve liver function following experimental hepatic injury, whereas higher doses can lead to hepatocyte damage. In this context, SeNPs have demonstrated hepatoprotective effects, preserving hepatic parenchyma and reducing liver enzyme levels. These findings suggest that therapeutic levels of selenium may exert beneficial effects, while higher concentrations may be detrimental [65].

The NCs cytotoxicity was also evaluated in two forms: (1) as a liquid GG polymer suspension before lyophilization (SeNPs@GG1) and (2) as a resuspended GG polymer suspension after lyophilization (SeNPs@GG2) (Figure 8d). The objective was to determine whether lyophilization affected the cytotoxicity of SeNPs. Both NCs were evaluated by MTT assay in HeLa cells at concentrations of 10–140 µg/mL to determine IC_50_ values. Results showed that SeNPs@GG1 and SeNPs@GG2 were more cytotoxic than SeNPs alone up to 70 µg/mL, likely due not only to NPs properties but also to GG itself, which may potentiate SeNPs toxicity. It has been reported that biopolymers may enhance the cytotoxic effects of metallic NPs, potentially by increasing medium viscosity, which can promote hypoxia-like conditions, and by interacting with membrane receptors to activate apoptotic signaling pathways [56]. Although GG is unlikely to be internalized by the cell due to its high molecular weight, in nanocomposite systems or in NP’s conjugates, GG may act as a coating that facilitates NPs’ internalization [66,67].

At concentrations between 90 and 140 µg/mL, both SeNPs@GG1 and SeNPs@GG2 displayed lower cytotoxicity than SeNPs, likely due to NPs’ encapsulation or reduced bioavailability when embedded within the polymeric matrix [68,69]. Statistically significant differences were also observed between SeNPs@GG1 and SeNPs@GG2 across 10–50 µg/mL, with SeNPs@GG1 exhibiting greater toxicity. This effect is probably due to improved dispersion, increased stability, and greater surface exposure of SeNPs in the gel-based polymeric network, leading to higher bioactivity [70]. Conversely, lyophilized NPs may aggregate or coalesce upon rehydration, thereby reducing cellular interactions and consequently, cytotoxicity [71]. IC_50_ values for SeNPs@GG1 and SeNPs@GG2 were calculated at 93.5 µg/mL and 93.7 µg/mL, respectively. These values are similar to those of SeNPs alone, indicating that the lyophilization and redispersion process did not significantly alter SeNPs’ cytotoxicity, supporting their suitability for controlled-release systems and therapeutic applications.

On the other hand, the viability of normal human primary endometrial cells was evaluated after exposure to SeNPs, SeNPs@GG1, and SeNPs@GG2 over a concentration range of 3.1–400 µg/mL, revealing a concentration-dependent decrease in viability across all treatments. Notably, cell viability remained near 80% at intermediate concentrations, whereas more pronounced reductions of ~30–40% were observed at 200 and 400 µg/mL. In contrast, these results differ markedly from those obtained in the HeLa cell line, where even the lowest concentration tested induced a significant decrease in viability. This difference is consistent with prior evidence indicating that cancer cells are more susceptible to nanomaterial-induced stress than non-cancerous cells. [72]. Collectively, these findings suggest a differential response between normal and cancer cells, highlighting the potential of SeNPs and NCs as selective agents for biomedical applications.

#### 2.3.2. Live/Dead Assay

**Figure 9:**
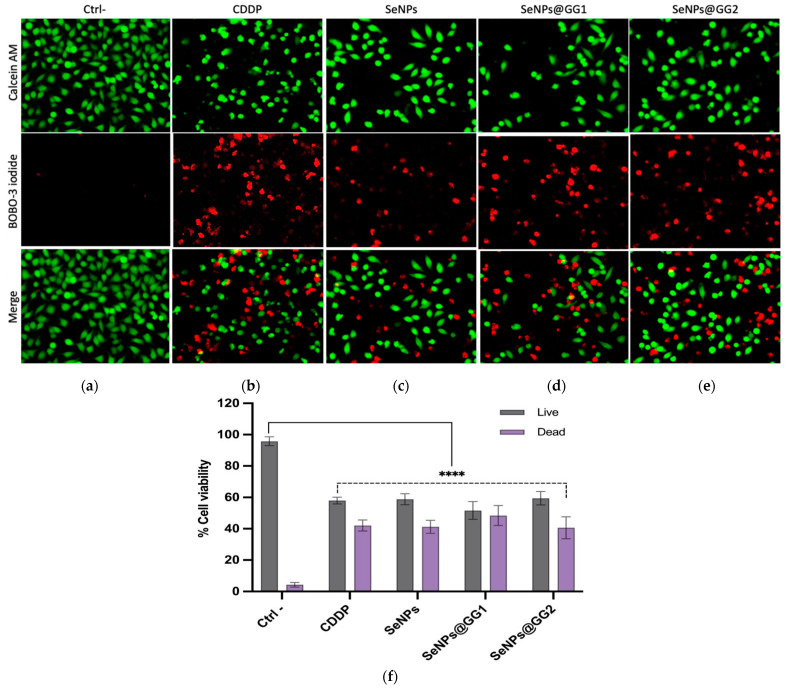
Live/Dead assay in HeLa cells (20×), after 24 h treatment at IC_50_ concentrations. (**a**) Negative control (Ctrl–), (**b**) CDDP, (**c**) SeNPs, (**d**) SeNPs@GG1, and (**e**) SeNPs@GG2. (**f**) Percentage of live and dead cells. Data are presented as mean ± standard deviation. Statistical significance was determined by two-way ANOVA (**** *p* < 0.0001). In this assay, viable cells are visualized in green and dead cells in red.

Once the IC_50_ values were established, a Live/Dead assay was performed to confirm the MTT cytotoxicity results through a fluorescence-based technique that detects cell membrane damage and loss of activity of hydrolytic enzymes such as esterases (Figure 9). The Live/Dead assay enabled visualization of both viable (green) and dead (red) cells and showed a consistent correlation with MTT results. The viability assay showed that 87% of AD-hMSCs were metabolically active, as indicated by the calcein-AM fluorescent signal after 15 days of culture, while 13% were reported as dead.

In the analyzed images, a significant reduction in viable cells was observed in most treatments relative to the negative control (Ctrl–), which showed over 90% viable cells (Figure 9a). For the antineoplastic agent CDDP, the proportion of live and dead cells was approximately 50% (Figure 9b). This trend was similar for the other treatments and was more evident in SeNPs@GG1 (Figure 9d) and SeNPs@GG2 (Figure 9e). The results showed strong agreement between the Live/Dead and MTT assays, as the percentages of viable and non-viable cells observed at the IC_50_ concentrations were consistent across both methods, further demonstrating the cytotoxic potential of the SeNPs (Figure 9c). Reports by Cruz et al. [73] showed similar findings using biosynthesized SeNPs from *E. coli* applied to lung cancer cells (A549), indicating that the Live/Dead assay can corroborate cytotoxic effects detected with the MTT assay. Additionally, their study demonstrated that SeNPs exerted potent cytotoxicity against cancer cells, including activation of caspase-3 and reduced cell proliferation, suggesting that apoptosis is the primary mechanism of cell death [73].

#### 2.3.3. Cell Morphology

**Figure 10:**
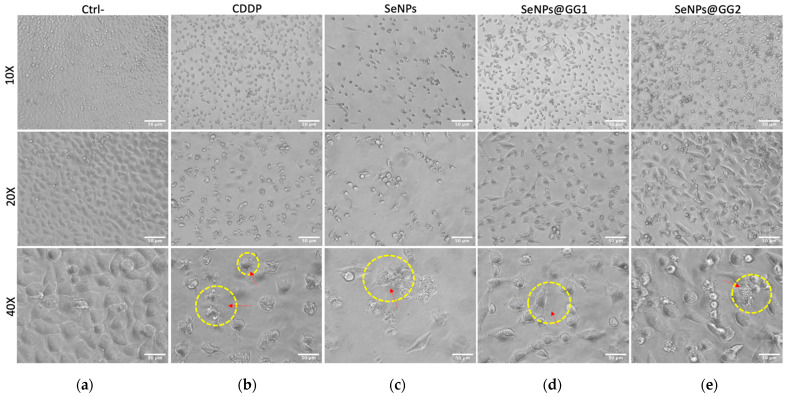
Representative images of morphological changes in HeLa cells after 24 h treatment at IC_50_ concentrations: (**a**) Ctrl–, (**b**) CDDP, (**c**) SeNPs, (**d**) SeNPs@GG1, and (**e**) SeNPs@GG2. Arrows and yellow circles indicate the major morphological changes observed in the treated cells (scale bar, 50 µm).

A morphological analysis of HeLa cells post-treatment was performed to evaluate structural, physiological, and plasma membrane integrity-related changes that may indicate cytotoxic effects, cellular stress, or cell death. The morphological evaluation of HeLa cells treated for 24 h with the IC_50_ of each treatment revealed significant alterations compared to the negative control (Ctrl–) (Figure 10a). Under all treatments, cell shape and size were altered, demonstrating a clear impact on cell morphological integrity. In CDDP-treated cells, significant morphological changes were observed, including roughened surfaces and apoptotic bodies, suggesting advanced apoptosis (Figure 10b). These findings are consistent with previous reports describing the cytotoxic action of CDDP, which does not directly target the plasma membrane. However, secondary cytotoxic effects may lead to membrane alterations, including phosphatidylserine externalization, formation of apoptotic bodies, and cell shrinkage [74].

Treatment with SeNPs also induced notable alterations (Figure 10c). Cells exhibited irregular morphologies, with some elongated and others shrunken, and a reduction in the number of adherent cells. Apoptotic bodies were evident, suggesting activation of cell death mechanisms potentially triggered by oxidative stress and mitochondrial dysfunction, as previously reported for similar NPs in related cell lines [75]. In the treatments with SeNPs@GG1 (Figure 10d) and SeNPs@GG2 (Figure 10e), the morphological effects were even more pronounced. In this case, cells displayed morphologies that were entirely distinct from the control, including elongation, shrinkage, rounding, loss of cellular architecture, and formation of structures associated with apoptotic bodies.

Additionally, characteristic morphological changes were observed in the treated cells, including the appearance of cytoplasmic projections resembling arm-like extensions and membrane blebbing, which are associated with cytoskeletal depolymerization and uncoupling of the plasma membrane from the actin cortex [76,77]. Filopodia were also identified, possibly arising from a cellular response to intracellular damage and the activation of apoptosis-related signaling pathways. These structures may also facilitate interactions between apoptotic cells and their microenvironment during the late stages of cell death. Furthermore, actin-rich filopodia have been described as a morphological hallmark of pyroptosis, aiding in their recognition by dendritic cells [78].

#### 2.3.4. Annexin V-FITC Apoptosis Assay

**Figure 11:**
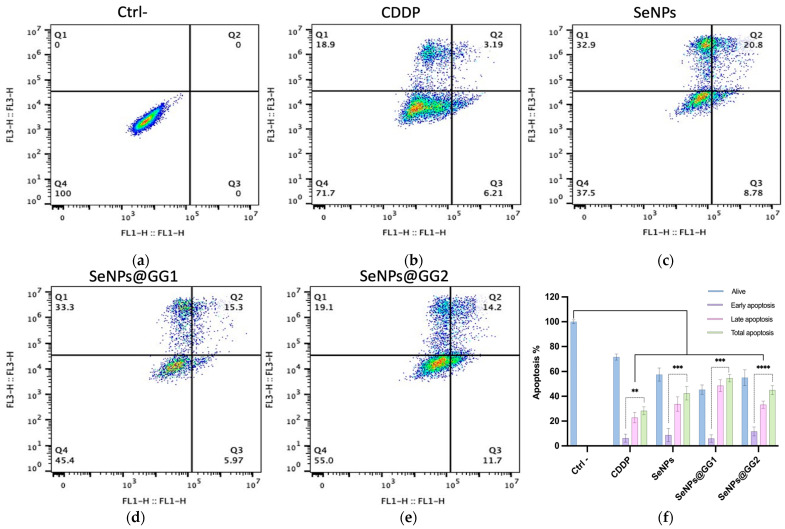
Apoptosis analysis by flow cytometry after 24 h treatment at IC_50_ concentrations: (**a**) Ctrl–, (**b**) CDDP, (**c**) SeNPs, (**d**) SeNPs@GG1, and (**e**) SeNPs@GG2. (**f**) Quantitative analysis of apoptotic populations. Data are presented as mean ± standard deviation. Statistical significance was determined by two-way ANOVA (** *p* < 0.01, *** *p* < 0.001, **** *p* < 0.0001).

To investigate the type of cell death induced in HeLa cells following treatment, apoptosis was assessed by flow cytometry (Figure 11). These analyses allowed the identification of cells undergoing early or late apoptosis, as well as necrosis, within the treated cell populations. Propidium iodide (PI) was used as a DNA marker to identify cells with compromised plasma membranes, characteristic of late apoptosis or necrosis. In contrast, cells with intact membranes but exhibiting phosphatidylserine (PS) externalization, indicative of early apoptosis, were detected by dual staining with PI and Annexin V-FITC.

Using the 24 h IC_50_ concentration for all treatments, the events in the quadrants suggest that in the negative control (Ctrl–), 100% of the cell population was in quadrant Q4, corresponding to viable cells (Figure 11a). In contrast, the data showed that CDDP-induced cell death was predominantly mediated by secondary apoptotic necrosis, consistent with CDDP’s known mechanism of action via apoptosis (Figure 11b). These findings are comparable to those reported by Mohiuddin and Kasahara [79], who observed that in A549 cells treated with CDDP, the highest percentage of cell death was in the necrotic fraction.

The results obtained for SeNPs (Figure 11c) and both NCs (Figure 11d,e) indicate that necrotic processes likewise mediated apoptosis. These observations are consistent with studies using green-synthesized SeNPs, which reported similar percentages of necrosis-associated apoptosis [80]. Furthermore, our findings are consistent with those of Cui et al. [81], who reported that SeNPs-induced apoptosis involved not only early apoptotic events but also a statistically significant proportion of necrosis. They further demonstrated the expression of pro-apoptotic proteins, including caspase-9, which activates caspase-3 and enhances apoptosis. Likewise, a decrease in Bcl-2 levels, a key anti-apoptotic protein in the mitochondrial pathway, was observed, leading to mitochondrial membrane permeabilization and the release of apoptosis activators, such as cytochrome C.

Additional apoptotic pathways associated with SeNPs exposure, beyond the classical p53- and AKT-dependent mechanisms [82], may involve mitochondrial apoptosis triggered by inhibition of EGFR-mediated and PI3K/AKT signaling and suppression of the Ras/Raf/MEK/ERK cascade, as well as activation of MAPK pathways [83]. Several studies also propose that autophagy is a SeNPs-induced cell death mechanism, associated with beclin-1 activation, p62 suppression, and inhibition of the PI3K/AKT/mTOR pathway. In HepG2 cells treated with laminarin-functionalized SeNPs (LP-SeNPs) for 12 h, increased LC3-II levels and decreased p62 levels were reported as key indicators of autophagy, along with LC3-I to LC3-II conversion and their recruitment to autophagosomal membranes [84,85]. Additionally, various selenium compounds, including SeNPs, have been shown to induce apoptosis in cancer cells through sustained endoplasmic reticulum (ER) stress [57].

One of the major mechanisms of SeNPs-mediated apoptosis in cancer cells is the overproduction of ROS, originating from mitochondrial electron transport chain dysfunction, which substantially contributes to cell death. Moreover, SeNPs may directly interfere with this signaling cascade, thereby enhancing cytotoxicity. SeNPs-induced apoptosis can be activated through two pathways: the extrinsic pathway, involving binding of pro-apoptotic ligands to death receptors and activation of caspase-8; and the intrinsic pathway, characterized by caspase-9 activation, typically in response to DNA damage or oxidative stress [86].

#### 2.3.5. ROS Assay

**Figure 12:**
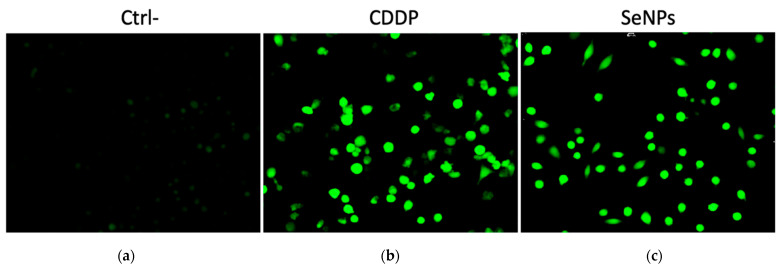

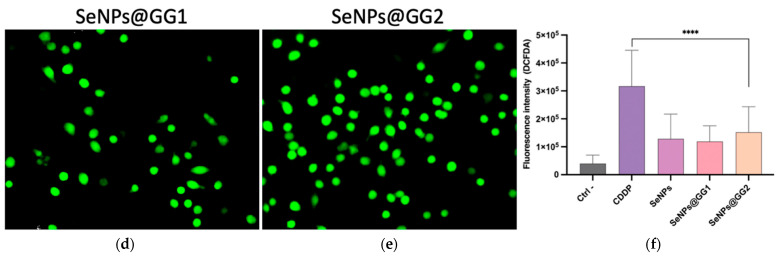
Reactive oxygen species (ROS) generation in HeLa cells after 24 h treatment at IC_50_ concentrations: (**a**) Ctrl–, (**b**) CDDP, (**c**) SeNPs, (**d**) SeNPs@GG1, and (**e**) SeNPs@GG2. (**f**) Quantitative comparison of intracellular ROS fluorescence intensity. Data are presented as mean ± standard deviation. Statistical significance was determined by one-way ANOVA (**** *p* < 0.0001). A significant increase in ROS production is shown in green.

The ROS assay using 2′,7′-dichlorofluorescein diacetate (DCFH-DA) was performed to measure intracellular ROS generated following treatment with CDDP, SeNPs, and NCs. The ROS analysis (Figure 12) revealed a substantial increase in ROS production (green) in treated cells compared with the Ctrl−.

The SeNPs@GG2 treatment (Figure 12e) showed the highest intracellular ROS levels, second only to CDDP (Figure 12b), the positive control. Due to its small molecular size, CDDP enters cells more readily, via passive diffusion and transporters CTR1 (copper transporter 1) and OCT2 (organic cation transporter 2) [87] than NPs-based systems, which mainly rely on clathrin-mediated endocytosis or caveolae-mediated endocytosis, depending on the specific receptor–ligand interactions initiated by the NPs.

However, there are also passively targeted NPs, whose internalization occurs primarily through pinocytosis or diffusion, driven by the size, shape, and surface charge of the NPs [88]. Once internalized, CDDP directly interacts with DNA and mitochondria, inducing oxidative stress and elevated ROS levels [87], whereas NPs-induced ROS production is highly dependent on physicochemical properties and cellular context [89]. Similar results were reported by Tang et al. [90], who demonstrated that SeNPs synthesized with ascorbic acid as the reducing agent and functionalized with betacyanins could induce intracellular ROS production, increasing by up to 136% at 10 μg/mL in the human hepatocellular carcinoma cell line HepG2. Additionally, other studies have shown that SeNPs can trigger a distinct form of programmed cell death, ferroptosis, which is iron-dependent and characterized by increased lipid ROS, depletion of GSH, reduced GPX4 expression, or altered intracellular iron levels [91].

#### 2.3.6. Mitochondrial Membrane Integrity Assessment

**Figure 13:**
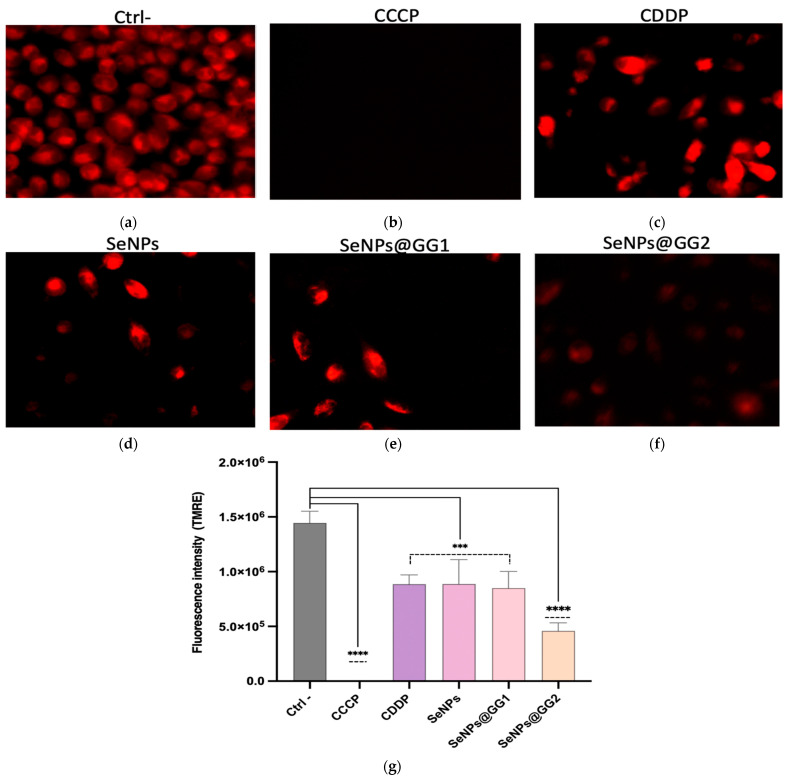
Mitochondrial membrane integrity assay after 24 h treatment at IC_50_ concentrations: (**a**) Ctrl–, (**b**) CCCP, (**c**) CDDP, (**d**) SeNPs, (**e**) SeNPs@GG1, and (**f**) SeNPs@GG2. (**g**) Quantitative comparison of intracellular TMRE fluorescence intensity. Data are expressed as mean ± standard deviation. Statistical significance was determined by one-way ANOVA (*** *p* < 0.001, **** *p* < 0.0001). Red color is associated with the integrity of the mitochondrial membrane in viable cells.

To further characterize the cytotoxic effects of the treatments, assays were performed to assess mitochondrial membrane integrity by detecting mitochondrial-associated fluorescence (red) in viable cells (Figure 13). The fluorescent dye tetramethylrhodamine ethyl ester (TMRE), which is cell-permeable and accumulates in intact mitochondria, was used along with the mitochondrial membrane potential disruptor carbonyl cyanide m-chlorophenyl hydrazine (CCCP) (Figure 13b). Depolarized or inactive mitochondria exhibited reduced fluorescence, reflecting decreased mitochondrial membrane potential and resulting in lower TMRE accumulation. Based on the ROS generation results, it can be inferred that excessive ROS production may alter membrane integrity.

The assay results revealed reduced accumulation of the TMRE compound in all four treatments compared with the negative control (Ctrl–), in which a high fluorescence intensity was observed, characteristic of cells with intact mitochondrial membranes (Figure 13a). No fluorescence was observed for CCCP (Figure 13b), as this mitochondrial uncoupler transports protons across the inner mitochondrial membrane, dissipates the electrochemical gradient, and collapses the mitochondrial membrane potential (Δψm), thereby directly impairing TMRE accumulation and promoting its release into the cytosol [92]. In the case of CDDP (Figure 13c), oxidative stress is one of the most important mechanisms involved in the drug’s toxicity, where mitochondria act as the primary target, resulting in the loss of mitochondrial integrity, depletion of mitochondrial protein sulfhydryl groups, inhibition of calcium uptake, and a reduction in mitochondrial membrane potential [74]. Regarding the SeNPs (Figure 13d) and SeNPs@GG1 (Figure 13e) treatments, a pronounced decrease in fluorescence and, consequently, in membrane integrity was observed, with the effect being most potent in SeNPs@GG2 (Figure 13f), a trend consistent with the results obtained in the ROS assay.

Several studies involving metallic NPs, such as gold NPs, indicate that nanometric materials may contribute to excessive ROS production, which is associated with mitochondrial membrane damage, directly compromising membrane potential and ultimately reducing ATP synthesis, leading to cell death processes such as apoptosis or necrosis [93]. Likewise, SeNPs have been shown to induce mitochondrial damage and, consequently, activate mitochondria-dependent apoptotic pathways, including those regulated by p53 and AKT. This process may also involve inhibition of the PI3K/AKT pathway mediated by the epidermal growth factor receptor (EGFR) and regulation of the Ras/Raf/MEK/ERK cascade. In addition, SeNPs can promote MAPK activation [94].

Furthermore, upregulation of p53 expression has been shown to promote its mitochondrial translocation, counteracting the anti-apoptotic effect of Bcl-2 [95]. Similarly, increased Bax expression and reduced Bcl-2 levels disrupt the balance between pro- and anti-apoptotic proteins, ultimately increasing mitochondrial membrane permeability. This shift facilitates the release of cytochrome c into the cytoplasm, a key event for apoptosome formation and subsequent activation of caspase-9, which then cleaves and activates caspase-3. The activation of this effector caspase represents a critical point in the execution phase of programmed cell death [57].

#### 2.3.7. Effect of SeNPs and NCs on DNA Damage

**Figure 14:**
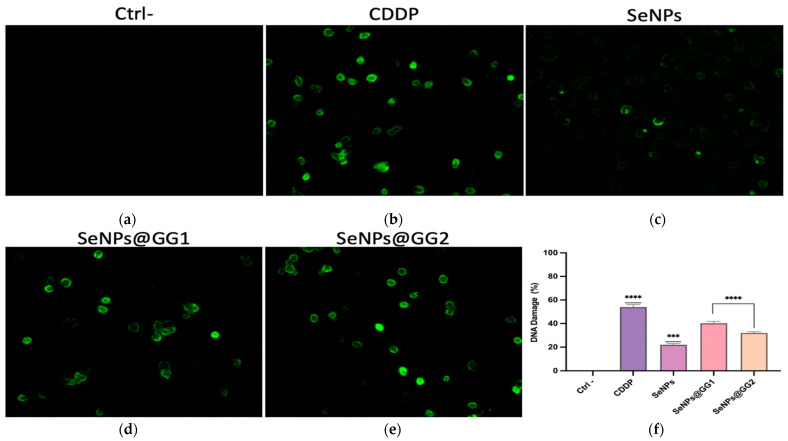
TUNEL assay for detection of DNA fragmentation in HeLa cells after 24 h treatment at IC_50_ concentrations: (**a**) Ctrl–, (**b**) CDDP, (**c**) SeNPs, (**d**) SeNPs@GG1, and (**e**) SeNPs@GG2. (**f**) Quantitative comparison of fragmented DNA fluorescence intensity. TUNEL-positive nuclei appear as bright green fluorescence (20× magnification). Data are presented as mean ± standard deviation. Statistical significance was determined by one-way ANOVA (*** *p* < 0.001, **** *p* < 0.0001). In this assay, nuclear damage is visualized in green, indicating DNA fragmentation.

To detect and quantify DNA damage induced by treatments with CDDP, SeNPs, and the NCs, a DNA fragmentation assay was performed using terminal deoxynucleotidyl transferase-mediated dUTP nick end labeling (TUNEL) (Figure 14). This technique revealed nuclear damage (green) in all treatments, including the CDDP-treated group, which showed the highest percentage of DNA fragmentation (Figure 14b). This finding is consistent with CDDP’s cytotoxic action. This alkylating agent covalently binds to purine bases (guanine and adenine), generating intra- and interstrand cross-links that disrupt DNA replication and transcription, ultimately leading to double-strand breaks [96].

All SeNPs (Figure 14c–e) treatments induced a significant increase in DNA fragmentation compared with the Ctrl– group (Figure 14a). Additionally, chromatin condensation was observed, a phenomenon characteristic of early apoptosis. This effect was particularly notable in the SeNPs@GG1 (Figure 14d) and SeNPs@GG2 (Figure 14e) treatments, suggesting greater apoptosis-inducing efficiency for this nanostructured system. The detected DNA fragmentation may be associated with increased ROS generation, a process frequently reported during cellular exposure to SeNPs [97].

The results from the ROS and mitochondrial membrane potential assays suggest that DNA fragmentation is mediated primarily by apoptotic nucleases, such as endonuclease G (EndoG) and caspase-activated DNase (CAD). Increased ROS levels induce oxidative stress, mitochondrial damage, and loss of mitochondrial membrane potential [98], promoting Bax- and Bak-mediated permeabilization of the outer mitochondrial membrane, a process negatively regulated by Bcl-2 and Bcl-xL [99]. This event allows the release of EndoG from the intermembrane space and its subsequent translocation to the nucleus. Once in the nucleus, EndoG first induces single-strand breaks in genomic DNA, which are subsequently converted into double-strand breaks, contributing to apoptosis in a caspase-independent manner [100].

CAD-mediated DNA fragmentation occurs during intrinsic mitochondrial or extrinsic apoptosis induced by cellular stress, such as increased ROS and mitochondrial depolarization [101]. These stimuli trigger mitochondrial permeabilization and the release of cytochrome c, which, together with Apaf-1 and procaspase-9, forms the apoptosome [101]. Caspase-9 activates caspase-3, which cleaves ICAD and releases CAD. CAD translocates to the nucleus, induces chromatin condensation, disassembly of nuclear lamina B, and double-strand breaks, generating oligonucleosomal fragments [102]. In parallel, γH2AX is massively recruited as a late marker of irreversible genomic degradation associated with caspase-dependent apoptosis [103]. The literature supports the cytotoxic and pro-apoptotic potential of SeNPs; for example, their ability to induce DNA fragmentation has been demonstrated in Caco-2 cells, as well as in A375 melanoma cells [104,105]. These findings are consistent with this study’s results and reinforce the potential of SeNPs as anticancer agents. Furthermore, the use of SeNPs stabilized with natural polymers, such as GG, not only improves their biocompatibility but may also enhance their controlled-release capacity, thereby optimizing cytotoxicity [18].

#### 2.3.8. Cell Cycle Analysis

**Figure 15:**
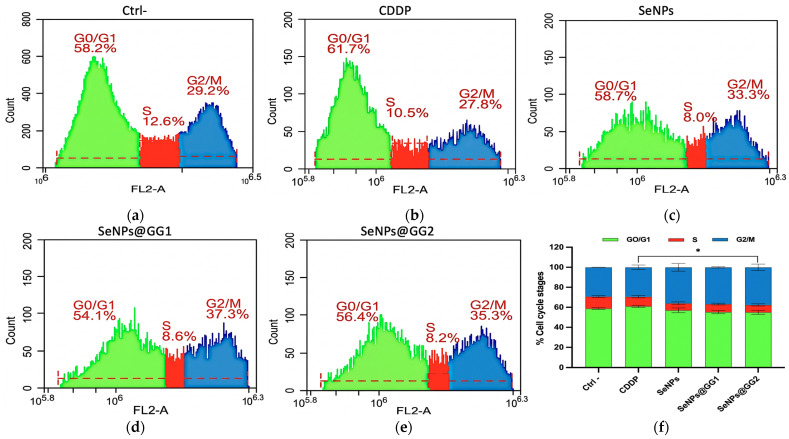
Flow cytometry analysis of cell cycle distribution in HeLa cells after 24 h treatment at IC_50_ concentrations: (**a**) Ctrl–, (**b**) CDDP, (**c**) SeNPs, (**d**) SeNPs@GG1, (**e**) SeNPs@GG2. (**f**) Quantitative distribution of cells in the G0/G1 (green), S (red), and G2/M (blue) phases. Data are presented as mean ± standard deviation. Statistical significance was determined by one-way ANOVA (* *p* < 0.05).

Cell cycle analysis was performed to assess cell distribution across the cell cycle phases and detect potential cell cycle arrest, DNA damage, or cell death. For this assay, HeLa cells were treated with the IC_50_ concentrations of the different compounds (Figure 15). A clear redistribution of cells across the cell cycle phases was observed in the treated groups, suggesting a cytostatic effect induced by all treatments.

Specifically, treatment with CDDP (Figure 15b) induced a predominant G0/G1 arrest, accompanied by a reduction in the proportion of cells in the S phase compared with Ctrl– (Figure 15a), consistent with its mechanism of action. CDDP exerts its antineoplastic effect by inducing DNA damage, activating cell cycle checkpoints, and halting progression at critical phases, such as G1 and G2, to allow DNA repair. However, when the arrest is prolonged or the damage is irreparable, cells may undergo apoptosis [106]. Previous studies have reported that in cancer cell lines such as HeLa and A2780 (ovarian cancer), CDDP can induce G0/G1 arrest depending on dose and exposure time [107,108], which has been linked to the activation of p53-dependent pathways that inhibit the transition to the S phase, interfering with cell cycle progression in cancer cells and contributing to its antitumor activity.

On the other hand, cells treated with pure SeNPs (Figure 15c), NCs SeNPs@GG1 (Figure 15d), and SeNPs@GG2 (Figure 15e) exhibited similar behavior, with a reduction in the percentage of cells in the S phase, suggesting inhibition of DNA synthesis. These results are consistent with previous studies in which SeNPs inhibited HeLa cell growth by inducing S-phase arrest [75]. Additionally, the percentage of cells in G2/M increased relative to the control, suggesting cell cycle arrest at this stage and a probable inhibition of cell proliferation in response to SeNPs-induced cellular stress. Therefore, the experimental results demonstrated that all treatments induced cytotoxicity in the HeLa cervical cancer cell line, suggesting that these compounds may serve as potential anticancer agents. Similar findings regarding the effect of SeNPs on the cell cycle have been reported previously, confirming their ability to induce arrest at different stages in various cancer cell lines, including MC-38 [108], HT29, Caco-2 [104], and A375 [105].

It has been reported that SeNPs induce DNA damage and activate cell cycle checkpoint pathways, leading to cell cycle arrest at different phases [109]. This effect is mainly associated with the activation of the ATM and ATR kinases, key regulators of the DNA damage response (DDR), as well as their downstream effectors Chk2 and Chk1, respectively, and the modulation of p53 [110]. The ATR/Chk1 pathway is predominantly activated in response to replication stress and single-strand DNA breaks (SSBs), promoting cell cycle arrest in the S and G2/M phases, stabilizing replication forks, and activating DNA repair mechanisms. In contrast, the ATM/Chk2 pathway responds to DNA double-strand breaks (DSBs), inducing arrest at the G1/S transition and regulating the activity of p53 and H2AX, proteins closely associated with DNA repair and the induction of apoptosis when the damage is irreparable [111,112].

Studies using metallic NPs demonstrated that they activate the ATM pathway in a dose-dependent manner, increasing ATM phosphorylation at Ser1981, p53 phosphorylation at Ser15, and Rad51 expression. These effects are associated with the generation of ROS, which mediate the activation of ATM kinase and other kinases, such as p38 MAPK and ERK. Additionally, H2AX phosphorylation was shown to depend on the activation of phosphatidylinositol 3-kinase (PI3K) family members, including ATM, ATR, and DNA-PK. Collectively, these findings suggest that nanoparticle-induced DNA damage is mediated through ROS-dependent activation of the ATM signaling pathway [113].

#### 2.3.9. Migration Assays

**Figure 16:**
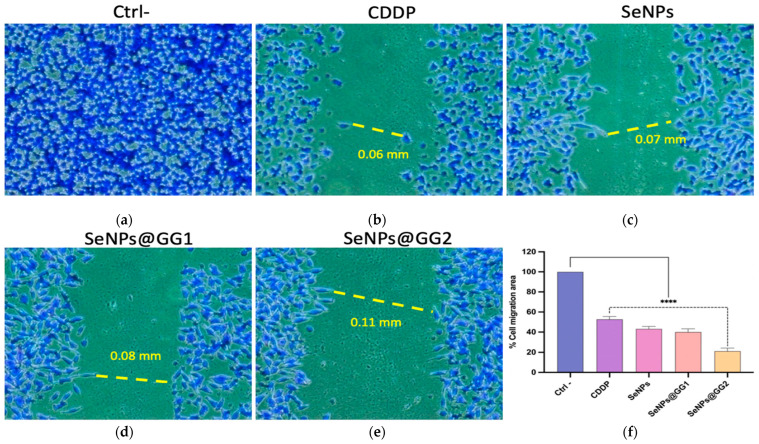
Cell migration assay in HeLa cells after 24 h treatment at IC_50_ concentrations, observed at 10× magnification: (**a**) Ctrl–, (**b**) CDDP, (**c**) SeNPs, (**d**) SeNPs@GG1, and (**e**) SeNPs@GG2. (**f**) Quantitative analysis of cell migration. Data are presented as mean ± standard deviation. Statistical significance was determined by one-way ANOVA (**** *p* < 0.0001).

The migration assay was used to evaluate the effects of CDDP, SeNPs, and NCs SeNPs@GG1 and SeNPs@GG2 on the migratory capacity of HeLa cells, using their respective IC_50_ concentrations over a 24 h period (Figure 16). Images captured at 10× showed that treatment with CDDP (Figure 16b) inhibited migration by 50%, consistent with previous studies reporting CDDP as a reducer of cell viability, migration, and invasion, and as a promoter of apoptosis in cervical cancer cells, possibly via negative regulation of the IL-17E/17RB signaling pathway [114].

In contrast, treatment with SeNPs (Figure 16c) resulted in only 42% cell migration, whereas SeNPs@GG1 (Figure 16d) and SeNPs@GG2 (Figure 16e) treated cells achieved 39% and 21% migration, respectively, both significantly lower than the complete migration observed in the negative control (Figure 16a). These statistically significant differences reflect the ability of SeNPs treatments to inhibit cell migration, which was more pronounced with SeNPs@GG2 (Figure 16f). These findings are consistent with the cell cycle results, which showed a reduction in the S phase across all SeNPs treatments, suggesting that reduced DNA synthesis could explain the reduction in cell migration capacity [115]. Other studies have reported similar results, showing that SeNPs inhibited proliferation in lung cancer cell lines (A549 and NCL-H23) [116].

**Figure 17:**
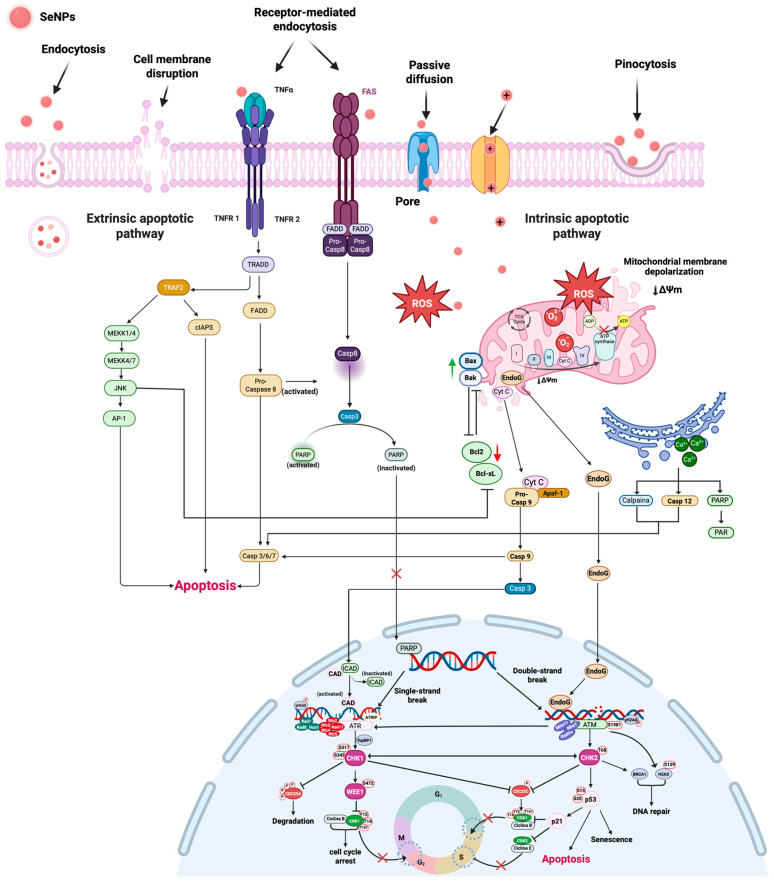
Schematic representation of the cellular mechanisms induced by selenium nanoparticles (SeNPs) leading to apoptosis. SeNPs enter cells via multiple routes, including endocytosis (receptor-mediated pathways such as TNFR/FAS), pinocytosis, passive diffusion, pore formation, or direct membrane disruption. Once internalized, they activate the extrinsic apoptotic pathway via TRADD/FADD recruitment and caspase-8 activation, which subsequently triggers executioner caspases (caspases-3/6/7). Figure created by the authors using BioRender.com. Agreement number: XC29MCUOMY.

Figure 17 illustrates the integration of the major known cellular mechanisms induced by selenium nanoparticles (SeNPs) that lead to apoptosis.

In parallel, SeNPs promote the generation of reactive oxygen species (ROS), activating the intrinsic apoptotic pathway characterized by mitochondrial membrane depolarisation (↓Δψm), modulation of Bcl-2 family proteins (↑Bax/Bak, ↓Bcl-2/Bcl-xL), and the release of cytochrome c and EndoG, leading to caspase-9 and caspase-3 activation. Additionally, increased intracellular Ca^2+^ levels and activation of calpains and caspase-12 further amplify apoptotic signaling.

ROS production and caspase activation also induce DNA damage (single- and double-strand breaks), triggering the ATM/ATR–CHK1/CHK2–p53 signaling axis, which results in cell cycle arrest, DNA repair, or apoptosis. PARP cleavage and CAD activation contribute to DNA fragmentation. Altogether, these processes integrate multiple signaling pathways that culminate in programmed cell death.

While the present study provided only an in vitro characterization that offers useful initial information, further studies under physiologically relevant conditions, including immunogenic responses, and an in vivo toxicological assessment, will be necessary to understand the system’s in vivo stability. SeNPs can undergo physicochemical transformations in biological environments, influenced by factors such as size, surface chemistry, and coating [117]. Moreover, SeNPs promote the activation of bone marrow-derived dendritic cells (BMDCs), stimulate IgG antibody production, facilitate the generation of toxin-neutralizing antibodies, and reduce pathological tissue damage, thereby garnering considerable interest as vaccine adjuvants due to their ability to significantly enhance total IgG responses [118,119]. Furthermore, in vivo studies have demonstrated that SeNPs exhibit dynamic biodistribution, accumulating in organs such as the liver, kidneys, spleen, and testes, showing dose-dependent effects. Toxicological assessments also report that evaluating the biological impact of SeNPs is complex and may include changes in metabolic and hepatic parameters [120,121].

## 3. Conclusions

In this study, a comparative physicochemical and cytotoxicity analysis of SeNPs and NCs (SeNPs@GG1 and SeNPs@GG2) was conducted on HeLa cells before and after lyophilization. The objective was to determine whether lyophilization affects the physicochemical and biological characteristics of SeNPs biosynthesized using an *A. glaucum* plant extract. The results demonstrated that SeNPs retained their properties after incorporation into the polymer matrix, lyophilization, and subsequent resuspension. UV-Vis spectroscopy detected a maximum absorption peak at 275 nm from the first 5 min of synthesis. It confirmed stability for at least 72 days in basic medium and sensitivity to acidic pH. FTIR analysis confirmed chemical interactions between the extract and sodium selenite, suggesting that the extract functions as a reducing and stabilizing agent. XPS analysis evidenced the formation of Se^0^ and other ionic species. The SeNPs exhibited an average size of 14 nm and a semi-spherical morphology, which was preserved after lyophilization. Although the matrix prevented direct observation of particles, homogeneous distribution was confirmed by Se mapping with EDS microanalysis. A defined nanocrystalline structure was also identified.

MTT cytotoxicity assays revealed that both SeNPs and the NCs (SeNPs@GG1 and SeNPs@GG2) reduced cell viability, with IC_50_ values of 98.3 µg/mL, 93.7 µg/mL, and 93.5 µg/mL, respectively, corroborated by Live/Dead analysis. The induced apoptosis was primarily associated with necrosis or late apoptosis. All treatments significantly increased ROS levels compared to the Ctrl–, altered cell morphology, decreased mitochondrial membrane integrity, and induced greater DNA fragmentation detectable by TUNEL (most pronounced in NCs). Additionally, a reduction in the proportion of cells in the S phase was observed, consistent with the migration assay, which demonstrated inhibited proliferation and reduced cell migration capacity. Overall, this study demonstrates the cytotoxic and pro-apoptotic activities of biogenic SeNPs, highlighting their potential as anticancer agents and as a possible addition to combination therapies. Further molecular studies, genotoxicity assays, and in vivo models are warranted.

## 4. Materials and Methods

Plant Material and Extract Preparation: *A. glaucum* leaves were collected in La Huerta, Jalisco (19°29′24.2″ N 105°02′33.9″ W), cleaned, frozen at −80 °C (Forma 900, ThermoFisher, Waltham, MA, USA), lyophilized for 5 days (Free Zone, Lab-conco, Kansas City, MO, USA), and ground in a mill (MF10BS1, Ika Werke, Wilmington, NC, USA). Extraction was performed by maceration in methanol (1:10, *w*/*v*) for 24 h, in triplicate. Methanol was used as an extraction solvent due to its high polarity and effectiveness in recovering a wide range of phytochemicals, particularly phenols and flavonoids, which play key roles in nanoparticle synthesis [6,122]. The solvent was then removed using a rotary evaporator (R-100, Buchi, Flawil, Switzerland) at 40 °C under reduced pressure. The *A. glaucum* leaf extract (AGLE) was stored at −80 °C, lyophilized, and then kept at room temperature.

Selenium Nanoparticles (SeNPs) Synthesis: SeNPs were synthesized via a green method adapted from Garza-García et al. [7]. A (5%) AGLE solution was added dropwise to (10 mM) Na_2_SeO_3_ (Sigma-Aldrich, St. Louis, MO, USA) while stirring at 1200 rpm for 40 min at 40 °C. The mixture was incubated in the dark at room temperature with constant agitation for 24 h. Finally, the SeNPs solution was frozen at −80 °C and lyophilized (LyoQuest, Telstar, Terrassa, Barcelona, Spain) for 24 h at −80 °C and 0.200 mBar. The dry product was stored at room temperature.

Nanocomposites (NCs) Preparation: Guar gum (GG) and SeNPs-based nanocomposites, SeNPs@GG1 (non-lyophilized liquid) and SeNPs@GG2 (lyophilized and resuspended), were obtained via freeze-drying. GG (1%) (Sigma-Aldrich, St. Louis, MO, USA) was dissolved in double-distilled water at 90 °C with stirring for 45 min, then allowed to rest at room temperature for 20 min. SeNPs (10 mg) were dispersed in 100 µL of double-distilled water and sonicated for 20 min at 45 °C. The SeNPs solution was then added dropwise to the GG solution under constant stirring at 45 °C for 30 min. The resulting gel was crosslinked for 20 min, frozen at −80 °C, and lyophilized for 24 h to prevent agglomeration and preserve the properties and efficacy of the NPs over long periods.

The reproducibility of SeNPs synthesis was assessed by preparing independent batches (*n* = 4) under identical conditions. Reaction yield was used as a primary indicator of batch-to-batch reproducibility to assess consistency across independent nanoparticle synthesis batches. The synthesis yield of SeNPs was evaluated using two complementary approaches: gravimetric yield and selenium-based real yield. After NPs’ formation, the suspensions were frozen and lyophilized, and the dry material was recovered and weighed using an analytical balance (ATX224, Cobos Precision, Barcelona, Spain).

The gravimetric yield was calculated as the ratio between the mass of the recovered lyophilized product and the initial mass of sodium selenite used as precursor, according to Equation (1):

(1) $$Gravimetric\;yield\;(\%)=100\times \frac{m\;lyophilized\;product}{m\;initial\;sodium\;selenite}$$

where m lyophilized product is the total dry mass obtained after lyophilization, and m is the initial mass of sodium selenite (Na_2_SeO_3_) added to the reaction.

To estimate the actual yield relative to the elemental selenium theoretically available from the precursor, the selenium-based real yield was calculated from the stoichiometric selenium content of sodium selenite. First, the theoretical mass of selenium was determined using Equation (2):

(2) $$m\;theorical\;Se=m\;initial\;sodium\;selenite\times \frac{M\;Se}{M\;sodium\;selenite}$$

where M Se is the atomic mass of selenium, and M Na_2_SeO_3_ is the molar mass of sodium selenite added to the reaction. Then, the selenium-based real yield was calculated using Equation (3):

(3) $$Real\;yield\;based\;on\;Se\;(\%)=100\times \frac{m\;lyophilized\;product}{m\;theoretical\;Se}$$

The mean yield and standard deviation were calculated to evaluate batch-to-batch variability. The coefficient of variation (CV, %) was also determined as an indicator of process consistency. All measurements were performed in four replicates.

### 4.1. Characterization of SeNPs

UV-Vis Spectroscopy: UV-Vis spectroscopy was used to standardize SeNPs synthesis and NCs preparation. The presence of SeNPs was confirmed by analyzing maximum absorption peaks between 200 and 500 nm using a NanoDrop spectrophotometer (NanoDrop 2000, Thermo Scientific, Waltham, MA, USA). Spectra were processed using OriginPro 2024.

Fourier Transform Infrared Spectroscopy (FTIR): Surface chemistry of SeNPs and NCs was analyzed via FTIR with attenuated total reflectance (FTIR-ATR) to identify functional groups in AGLE potentially involved in SeNPs formation and stabilization (TENSOR II, Bruker Optics, Billerica, MA, USA) in the 4000–400 cm^−1^ range.

X-ray photoelectron spectroscopy (XPS) measurements were conducted to analyze the surface composition to determine the chemical environment of SeNPs using a Phoibos 150 detector and a monochromator Al Kα (1486.7 eV) source (Specs, Berlin, Germany). Samples were irradiated and measured at 6.6 × 10^−9^ mbar of vacuum in the chamber, taking 15 scans for each core level with 20 eV of pass energy and 0.1 eV of step size, for high-resolution spectra.

Transmission Electron Microscopy (TEM): SeNPs size and morphology were assessed using TEM (JEM-2100, JEOL, Tokyo, Japan) at 200 kV with a Gatan OneView 4K camera (Gatan, Inc., Pleasanton, CA, USA).

Scanning Electron Microscopy (SEM) and EDS: NCs morphology was analyzed by high-resolution SEM (JSM-IT300, JEOL, Tokyo, Japan). Samples were mounted on carbon tape, coated with gold, and observed at 15–20 kV with magnifications from 100× to 50,000×. Elemental composition and Se distribution were determined by energy-dispersive X-ray spectroscopy (EDS) coupled to SEM.

X-ray Diffraction (XRD): The crystalline structures of SeNPs and NCs were analyzed using an Empyrean diffractometer (Malvern Panalytical, Worcestershire, UK) in 2θ mode from 10° to 90°, with a current of 35 mA, a voltage of 40 kV, and a scanning rate of 0.01°/s.

### 4.2. Cytotoxicity Assessment of SeNPs and NCs

Cell Culture: Cytotoxicity assays were performed using human epithelial adenocarcinoma HeLa cells (ATCC^®^ CCL-2, American Type Culture Collection, Manassas, VA, USA) and normal human primary endometrial cells (obtained from healthy donors and supplied by the Centro de Biotecnología Santer (Guadalajara, Mexico), following institutional ethical guidelines and informed consent. Cells were cultured in T75 flasks (Corning, NY, USA) in DMEM (Biowest, Nuaillé, France) with 10% FBS (Gibco, Waltham, MA, US) and (1%) PSN antibiotics (Sigma-Aldrich, St. Louis, MO, USA) at 37 °C, 80% humidity, 5% CO_2_ (Thermo-Fisher 1168751, Waltham, MA, USA).

MTT Viability Assay: Cell viability was assessed using the MTT colorimetric assay, which measures enzymatic reduction in tetrazolium to formazan. HeLa cells were exposed to cisplatin (CDDP) (Intas Pharmaceuticals, Ahmedabad, Gujarat, India), SeNPs, and NCs for 24 h, then incubated with (5 mg/mL) MTT at 37 °C for 3 h. Formazan was dissolved in DMSO, and the absorbance was measured at 570 nm. IC_50_ values were calculated using non-linear regression in GraphPad Prism 9 software (GraphPad Software Inc., San Diego, CA, USA).

Live/Dead Assay: Cell viability was analyzed with the LIVE/DEAD^®^ Cell Imaging Kit 488/570 (Thermo Fisher, Waltham, MA, USA), which stains viable cells green (calcein-AM) and dead cells red (BOBO-3). HeLa cells were treated with CDDP (cisplatin), SeNPs, and NCs at IC_50_ concentrations for 24 h, then stained and imaged at 20× magnification under a fluorescence microscope (Optika, Ponteranica, Bergamo, Italy).

Flow Cytometry for Apoptosis: Early apoptosis was analyzed using the Annexin V-FITC Kit (Cell Signaling Technology, Danvers, MA, USA). A total of 1 × 10^5^ HeLa cells were treated with IC_50_ concentrations of CDDP, SeNPs, and NCs for 24 h, trypsinized, washed, stained with Annexin V-FITC and PI, and analyzed by flow cytometry (BD Accuri C6 Plus, Franklin Lakes, NJ, USA). Results distinguished viable, early apoptotic, and late apoptotic cells.

Reactive Oxygen Species (ROS) Levels: Intracellular ROS levels were assessed using DCFH-DA staining. HeLa cells treated with IC_50_ concentrations of CDDP, SeNPs, and NCs for 24 h were stained and imaged using blue fluorescence filters (Optika, Ponteranica, Bergamo, Italy).

Mitochondrial Membrane Potential: Mitochondrial membrane potential was assessed using the Mitochondrial Membrane Potential Assay Kit II (#13296, Cell Signaling Technology, Danvers, MA, USA) with TMRE and CCCP as a mitochondrial uncoupler. Cells were treated for 24 h with IC_50_ concentrations and imaged by fluorescence microscopy (Optika, Ponteranica, Bergamo, Italy).

Cell Morphology: HeLa cells were seeded at 1 × 10^4^ cells/well in 96-well plates, treated at IC_50_ concentrations, incubated for 24 h, and observed under inverted optical microscopy (Optika, Italy) to assess morphological alterations.

DNA fragmentation Assay: DNA fragmentation was assessed by TUNEL using a fluorescent kit (488 nm, Cell Signaling Technology, Danvers, MA, USA). Cells were treated for 24 h with IC_50_ concentrations of CDDP, SeNPs, SeNPs@GG1, and SeNPs@GG2, fixed with 4% formaldehyde, permeabilized, incubated with TUNEL reaction mix, and analyzed under a fluorescence microscope (Optika, Ponteranica, Bergamo, Italy).

Cell Cycle Analysis: HeLa cells (1 × 10^5^/well) were treated with IC_50_ concentrations of CDDP, SeNPs, and NCs for 24 h, trypsinized, stained with PI, and analyzed by flow cytometry (BD Accuri C6 Plus, Franklin Lakes, NJ, USA) using the FL2-A channel.

Migration Assay: A total of 1 × 10^5^ cells/well were seeded in 24-well plates and incubated for 24 h. Wounds were created using a 10 µL tip, washed with PBS, and treated with IC_50_ concentrations for 24 h. Cells were fixed in cold MeOH for 10 min, stained with 100 µL of crystal violet (Sigma-Aldrich, St. Louis, MO, USA) for 10 min, washed, dried, and imaged at 10× (Optika, Ponteranica, Bergamo, Italy).

Statistical Analysis: SeNPs characterization data were analyzed using OriginPro 2024. One-way and two-way ANOVA were used to analyze cytotoxicity data. Data are presented as mean ± standard deviation. Differences were determined using Duncan’s multiple range test with significance at *p* < 0.05 (Statgraphics, GraphPad Prism 9).

## Data Availability

The original contributions presented in this study are included in the article. Further inquiries can be directed to the corresponding authors.
